# Almond protein and whey protein beverages elicit comparable appetite regulation, gastrointestinal symptoms and hydration responses in postmenopausal women: a randomised crossover trial

**DOI:** 10.3389/fnut.2026.1918989

**Published:** 2026-07-28

**Authors:** Charles Urwin, Vy Tran, Michael Tieland, Sze-Yen Tan, Jackson Fyfe, Simon Feros, Rhiannon Snipe, Giselle Allsopp, Clinton Bruce, Greg Kowalski, Shaun Mason, Amelia Carr, Gavin Abbott, Zoya Huschtscha, Lee Hamilton

**Affiliations:** 1 Faculty of Health, School of Exercise and Nutrition Sciences, Institute for Physical Activity and Nutrition, Deakin University, Geelong, VIC, Australia; 2 Metabolic Research Unit, Faculty of Health, School of Medicine, Institute for Physical Activity and Nutrition, Deakin University, Geelong, VIC, Australia

**Keywords:** almond protein, appetite, dietary fibre, dietary supplementation, hydration, plant-based, postmenopausal women

## Abstract

**Introduction:**

Postmenopausal women experience physiological changes in appetite regulation, gastrointestinal function and hydration that may influence utility of protein supplements. Almond protein powder (APP), a plant-derived by-product of almond oil extraction, provides protein alongside fibre and unsaturated fat, which may influence these outcomes differently from whey protein (WP). Objectives of this study were to compare acute appetite, gastrointestinal and hydration responses to APP, WP, placebo and water ingestion in postmenopausal women.

**Materials and methods:**

Nine postmenopausal women (60 ± 4 y) completed a randomised crossover trial involving four treatments: APP (29.5 g protein), WP (29.5 g protein), placebo and water. Appetite and gastrointestinal symptoms (GIS) were assessed over 180 min using visual analogue scales. Hydration outcomes included urine colour, urine specific gravity, urine and plasma osmolality, fluid balance and beverage hydration index (BHI). Outcomes were analysed using linear mixed models with significance accepted at *p* < 0.05.

**Results:**

APP and WP suppressed appetite relative to baseline and water during the first 60 min post-ingestion period. Appetite area under the curve differed significantly between water and all other treatments (all *p* < 0.001), with APP, WP and placebo associated with lower appetite than water. GIS burden was low across all treatments, with no severe symptoms reported. Hydration markers demonstrated expected temporal changes; early dilution followed by progressive reconcentration. Significant treatment effects were observed for several hydration variables, including urine colour (*p* = 0.005), fluid balance (*p* < 0.001) and BHI (*p* = 0.017). Post-hoc comparisons identified differences between water and each nutrient-containing beverage for most hydration markers.

**Conclusion:**

APP and WP elicited comparable acute appetite suppression, gastrointestinal tolerance and hydration responses in postmenopausal women. While all nutrient-containing beverages reduced appetite relative to water during the early post-ingestion period, no meaningful differences were observed between APP and WP across appetite, GIS or hydration outcomes. These findings suggest that APP may represent a well-tolerated plant-based alternative to WP for protein supplementation in postmenopausal women. However, given the small sample size and acute nature of the intervention, larger and longer-term studies are required to determine whether these responses translate to habitual use and clinically relevant health outcomes.

**Clinical trial registration:**

Identifier 2024/HE000669; ACTRN12625000127404p. https://www.anzctr.org.au.

## Introduction

Menopause is associated with adverse changes in body composition, musculoskeletal health and cardiometabolic function, increasing the importance of nutritional strategies that support healthy ageing in postmenopausal women (1–7). Protein supplementation may help attenuate age-related declines in muscle mass and physical function, while also influencing appetite regulation and hydration status. At the same time, consumer interest in plant-based protein sources has increased substantially in recent years due to environmental, ethical and health-related motivations (8, 9). As a result, plant-derived protein supplements are increasingly being explored as alternatives to traditional animal-based proteins such as whey protein (WP), to support dietary frameworks that prioritise whole food approaches to adequate protein intake to prevent sarcopenia (10, 11). Almond protein powder (APP), commonly produced as a by-product of almond oil extraction, contains protein alongside fibre and other nutrients that may influence digestion, satiety and fluid retention differently to WP (12, 13). In contrast, WP is rapidly digestible, low in fibre and widely used as a reference protein supplement in nutrition research (14, 15). These differences in nutrient composition may alter appetite-related perceptions and hydration responses following ingestion.

Protein intake is consistently associated with greater suppression of hunger and increased feelings of fullness compared with lower-protein comparators (16–20). These effects may be particularly relevant during and after menopause, when endocrine changes can disrupt appetite regulation (21, 22). In addition, dietary fibre and beverage characteristics such as viscosity and volume may further influence satiety and thirst perceptions (23–26). Understanding these responses is important, as appetite and thirst-related perceptions may influence habitual dietary and fluid intake behaviours in postmenopausal women.

Gastrointestinal tolerability is also an important consideration for the suitability of protein supplements in older populations. Ageing is associated with changes in gastrointestinal function that may alter tolerance to certain foods (27), while symptoms such as bloating or abdominal discomfort may reduce adherence to supplementation strategies (28–31). Hydration responses are similarly relevant, given age and menopause-related changes in fluid regulation and thirst perception (32–36). Even mild dehydration may negatively affect physical and cognitive function (37), highlighting the importance of understanding how different protein beverages influence fluid balance. Assessing multiple hydration indices, including urine output, fluid retention, urine specific gravity (USG), osmolality and plasma volume change (PVC), provides a more comprehensive evaluation of whole-body fluid handling following beverage ingestion than any single marker alone (38). The Beverage Hydration Index (BHI) standardises comparisons between beverages by quantifying fluid balance relative to water, allowing hydration effects attributable to differences in nutrient composition to be assessed (39).

Accordingly, the aim of this study is to compare the acute effects of APP, WP, placebo and water on appetite perceptions in postmenopausal women, with additional assessment of gastrointestinal symptoms, tolerability and hydration. It is hypothesised that APP and WP will produce similar appetite and gastrointestinal responses, while APP may promote greater fluid retention and hydration due to its fibre content.

## Materials and methods

A detailed version of the proposed study protocols were published elsewhere (40). Any deviations to the originally proposed protocol have been disclosed and discussed here. This study was registered *a priori* with ANZCTR (ACTRN12625000127404p).

### Study design and setting

This randomised crossover study was conducted at Deakin University (Melbourne, Australia). The crossover design allowed rigorous assessment of hydration and perceived responses in postmenopausal women following ingestion of APP, whey protein, placebo or water. The SPIRIT 2013 statement was used in designing this study protocol (41); the CONSORT 2025 checklist was used in reporting results in this manuscript (42). All testing occurred between July 2025 and February 2026.

### Inclusion and exclusion criteria

Participants were eligible if they met each of the following criteria; biological female at birth, aged 50–65 years old, had undergone menopause (no menstrual bleeding for at least 12 consecutive months), body mass index between 18 and 35 kg.m^2^, stable body mass for the prior 2 months (not exceeding a change of 2 kg), stable use or non-use of hormone replacement therapy for the prior 6 months. Individuals were excluded from participation if they presented any of the following criteria; known nut allergy, current smoker, adherence to a vegan diet, current supplementation with protein powder, current chronic disease of gastrointestinal or metabolic nature (full list reported in the protocol paper).

### Sample size and recruitment

Sample size calculations were performed using Stata SE18 (StataCorp, Texas, United States) based on previously published data in older adults or women (43). The full *a priori* methodology was published previously (40). A minimum sample size of nine participants was calculated to provide sufficient statistical power to detect differences in amino acid concentrations, a primary outcome of the broader project with those data to be reported separately, a sample size that is also consistent with similarly designed studies in older or postmenopausal adults that assess subjective ratings of appetite (44). Participants were recruited using a multi-modal approach, using social media and local community networks. Interested individuals were provided with a plain language statement, which outlined study aims and prospective risks and benefits of participation.

### Interventions, blinding and order randomisation

Participants consumed one of four treatments in each experimental testing session:

1. APP – almond protein powder (56 g, provided by Harriss Woolf Almonds, California, United States), mixed with chocolate flavouring and almond milk (total protein content = 29.5 g, Table 1).
2. WP – whey protein powder (flavourless, 30 g of powder, purchased from Bulk Nutrients, Tasmania, Australia), mixed with chocolate flavouring and almond milk (total protein content = 29.5 g).
3. Placebo – maltodextrin (flavourless, 60 g of powder, purchased from Bulk Nutrients, Tasmania, Australia), mixed with chocolate flavouring and almond milk (total protein content = 3.3 g).
4. Water only (300 mL, total protein content = 0 g).

**Table 1:** Macronutrient, fibre, sodium and energy content of two protein drinks and two comparator treatments.

Dietary component	Treatment composition			
	APP	WP	Placebo	Water
Carbohydrate (g)	43.2	27.5	82.5	0.0
Protein (g)	29.5	29.5	3.3	0.0
Fat (g)	12.5	5.5	5.2	0.0
Fibre (g)	13.0	3.0	3.0	0.0
Sodium (mg)	202	261	198	9.0
Energy (kJ)	1724	1,210	1,689	0.0
Fluid mass (g)	300	300	300	300

Almond protein powder (APP) and whey protein (WP) consist of the relevant protein powder, almond milk and chocolate flavouring at the quantities listed. Placebo consists of almond milk, maltodextrin and chocolate flavouring at the quantities listed. Water consists of water only. All values are sourced from manufacturer-reported nutrition information statements. Fluid mass for the first three treatments was comprised of almond milk only. Harris Woolf supplied APP on behalf of the Almond Board of California. Each of APP, WP and placebo contained 30 g of chocolate flavouring and 300 g of almond milk. To standardise protein content, APP contained 56 g of powder and WP contained 30 g of powder. Placebo contained 60 g of maltodextrin. NB: full specific composition of each ingredient is presented in the protocol paper.

In each of the APP, WP and placebo treatments, 30 g of chocolate powder and 300 mL of almond milk (both Coles branded and purchased from Coles Supermarkets, Australia) were mixed with the powder in question to match protein content. Maltodextrin quantity was established to match energy content with APP, as part of an affiliated study (45). Drinks were divided into two equal parts (150 mL each) and stored at 4 °C. Participants consumed the first part within five minutes, rested for five minutes, then consumed the second part within an additional five minutes. Full details of the macronutrient content and other characteristics of the experimental treatments are presented in Table 1, with additional details also reported in Supplementary File 1.

Order of treatment administration was randomised using a Williams Design (46) with modifications (sequence reversals) to maximise the number of unique sequences. Full randomisation processing is outlined in the protocol paper. Participants were partially blinded, given the water treatment could not replicate the other three treatments. The supplement belief questionnaire was used to assess the efficacy of blinding.

### Testing session overview

**Figure 1:**
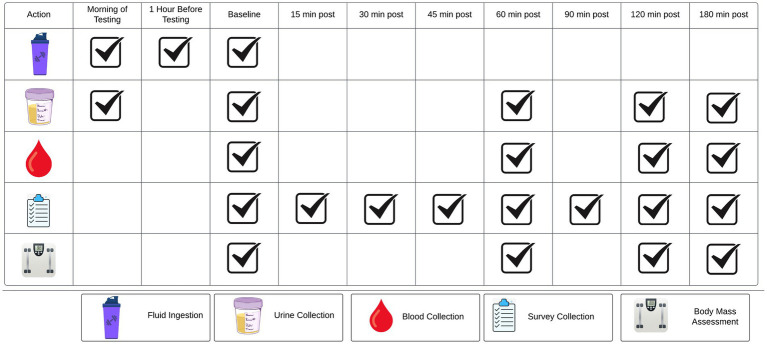
Schematic of experimental testing session overview, illustrating the data collection processes completed during each of the four treatments.

An overview of experimental testing sessions is presented in Figure 1, drawn from the protocol paper. Of note, the overview presented in Figure 1 has been adapted to reflect only collection of blood samples that are analysed for markers presented in this manuscript, with some unrelated markers to be presented in an affiliated manuscript (45). Briefly, four experimental testing sessions were completed by each participant. Each session followed the same protocol, with only the experimental treatment differing across sessions.

Participants arrived at each session after completing an overnight fast from 22:00 h the night before (water excepted). Participants replicated and recorded their diet and exercise for the 24 h prior to each session using EasyDietDiary (Xyris Software; Brisbane, Australia) during which time they avoided alcohol intake and sauna use. Participants were instructed to consume 500 mL of water in the hour prior to arriving at the laboratory, to ensure euhydration at the commencement of each session. During testing sessions, participants remained seated except when attending the bathroom for urine excretion or for collection of body mass measurements. A washout period of at least 48 h was enforced between trials, to allow recovery of any bruising at the sites of blood collection, and to ensure full return to basal state after the transient and acute responses expected following ingestion of the experimental treatments.

### Measurement procedures

#### Questionnaires and other subjective measures

Subjective ratings of hunger, fullness, desire to eat and urge to eat were assessed at baseline, and then 15, 30, 45, 60, 90, 120 and 180 min after ingestion of experimental treatments using visual analogue scales anchored at 0 and 50 (VAS). Gastrointestinal symptoms were assessed at the same times, using a Likert-type scale (47). Participants rated the extent to which they liked drinking the supplement, and the extent to which they liked the taste of the supplement, both rated from 1 = not at all, to 7 = extremely. Participants also reported the extent to which they would take this supplement again, on a scale ranging from 1 = I would drink this food every opportunity I had, to 9 = I would drink this only if I were forced to (note: a score of 10 was possible, but is described as: I have never tried this, so was deemed unlikely to be reported in the post ingestion period of this study). This was completed in the 15 min after ingestion of the experimental treatment. Participants completed questionnaires assessing the extent to which they could identify the treatment they had ingested, and the confidence with which they could do so on a VAS (supplement belief questionnaire). Where participants reported that they could not identify which treatment they had ingested, they did not report any confidence score. These two items were completed both immediately post-ingestion, and at the end of each testing session. All questionnaires were attached to the protocol paper as Appendices.

#### Blood and urine collection

Blood samples were collected via intravenous cannulation from the antecubital vein. After two baseline samples were drawn (mean values reported), blood samples were subsequently collected every 60 min following supplement ingestion (60, 120, 180 min). All samples were drawn to approximately 2 mL volume, into lithium-heparin coated vacutainers. After aliquots were drawn for assessment of haematocrit and haemoglobin, remaining whole blood was centrifuged at 3600 RPM for 10 min at 4° C, with plasma then immediately analysed for osmolality (Osmometer K-7400S, Knauer). Participants voided their bladder at baseline, 60, 120 and 180 min after supplement ingestion to assess hydration indices of mass and contents (voiding occurred prior to other measurements collected at the same time point). Where participants could not void any urine, no measurements were possible. Where participants requested voiding their bladder at a time other than those listed above, assessment of that urine was performed at the next planned time point (e.g., a participant collected urine at 85 min post ingestion, this was stored and then assessed at 120 min).

#### Body mass assessment

At baseline only, bioelectrical impedance analysis (BIA) was used to assess simple indices of body composition. BIA outcomes are acknowledged to be susceptible to fluid intake, hence the use of this measurement at baseline only. At baseline, and then every hour after supplement ingestion, body mass was assessed using standard laboratory scales, sensitive to 50 g (body mass scales, IC-UC-321; A&D Co., Ltd.; Tokyo, Japan).

### Outcome measures

#### Questionnaires and other subjective measures

Values obtained from the VAS for hunger, fullness, desire and urge to eat were recorded as a numerical value from 0 to 50, with higher values indicating stronger perception of that outcome. A composite appetite score was calculated by summing the scores for hunger, desire to eat and urge to eat with the inverse of the fullness score (i.e., 50 minus the observed fullness value). The maximum possible value for the composite score at each time point was therefore 200, where a higher value corresponds to a state of greater overall appetite. Individual and composite responses were quantified as incremental area under the curve (iAUC) relative to baseline, commencing immediately after ingestion, using the trapezoidal method. iAUC was calculated as a net incremental area, such that values above and below baseline contribute positively and negatively, respectively. The ratings of 16 individual GIS were summed to generate a single score at each time point (seven upper GIS, six lower GIS, three other GIS). The cumulative total rating of all symptoms across entire sessions after ingestion was assessed as a discrete variable. Time course analyses were compromised by the substantial proportion of GIS values equaling a score of 0. Any GIS rated at a value ≥5 out of 10 was classified as severe. The frequency of GIS was also reported as a % of sessions and a % of time points where symptoms were reported, regardless of the severity of symptoms. The accuracy with which participants identified the treatment they had been administered was reported as a % of sessions where the correct treatment was identified (completed 15- and 180-min post-ingestion). Confidence of this identification was also reported as a discrete variable, with values reported on a 0–50 VAS. The scores obtained from participants rating the likability of the supplement were assessed as discrete variables.

#### Blood, urine and body mass data

All blood samples were assessed for haematocrit and haemoglobin (EKF Diagnostics Holdings plc, Cardiff, Wales, United Kingdom), which were used to calculate relative plasma volume change percentage from baseline (PVC %) using equations derived previously by Dill and Costill (48). PVC values were handled as time course variables in statistical analyses, to be described in subsequent sections. Plasma was measured for osmolality, with time course outcomes assessed. All urine specimens were assessed for urine specific gravity (USG, ratio PAL-10S; ATAGO Co., Ltd.; Tokyo, Japan), urine colour via eight-point visual scale [AU, (49)], and osmolality. Urine samples collected post-ingestion were also assessed for excreted mass (g, MII-2500; Universal Weight Electronics; Geldermalsen, Netherlands). Fluid balance (mass and percentage) was determined via calculation of urine mass subtracted from fluid mass ingested (300 g in all treatments). Beverage Hydration Index (BHI) was calculated according to previously published methods pioneered by Maughan and colleagues (39), as the ratio of cumulative urine mass following ingestion of water to that following ingestion of each experimental drink over the same time. Values exceeding 1.0 indicate greater fluid retention relative to water. BHI was calculated at 120 min to replicate Maughan et al.’s timing, and at 180 min to reflect the end point of the present experimental sessions. Outcomes related to body composition assessed using BIA were body mass (kg), total body water (kg) and body fat percentage (%), collected at baseline only. Body mass (kg) was collected at baseline, and then 60, 120 and 180 min post-ingestion using scales mentioned previously.

### Statistical analyses

All analyses were performed using Stata SE18 (StataCorp, Texas, United States), with statistical significance set at *p* < 0.05. Normality was assessed for each discrete variable using Shapiro–Wilk tests (*p* < 0.05 indicating violation). Statistics are presented as mean ± SD for normally distributed variables and median (range) for non-normally distributed variables. Normally distributed outcomes from blood and urine sample collection were analysed using linear mixed models (LMMs) with treatment and session number as fixed effects and participant as a random intercept. Overall treatment effects were assessed using joint Wald tests, with estimated marginal means and Bonferroni-adjusted pairwise comparisons for post-hoc analyses.

Appetite outcomes (hunger, fullness, desire to eat, urge to eat and appetite score) were analysed using generalised LMMs with a gamma distribution and log link to account for positive skew (due to large number of 0 s reported). A small constant (0.01) was added to all 0 values for hunger, fullness, desire to eat and urge to eat to enable model convergence and avoid undefining log-linked values. Fixed effects included treatment, time, and their interaction, with session included as a fixed effect and participant as a random intercept. Overall treatment, time, and interaction effects were assessed using joint Wald tests. Marginal predicted means were back transformed for presentation, and pairwise comparisons adjusted for multiple comparisons were conducted to assess differences between treatments at each time, and between times within treatments.

**Figure 2:**
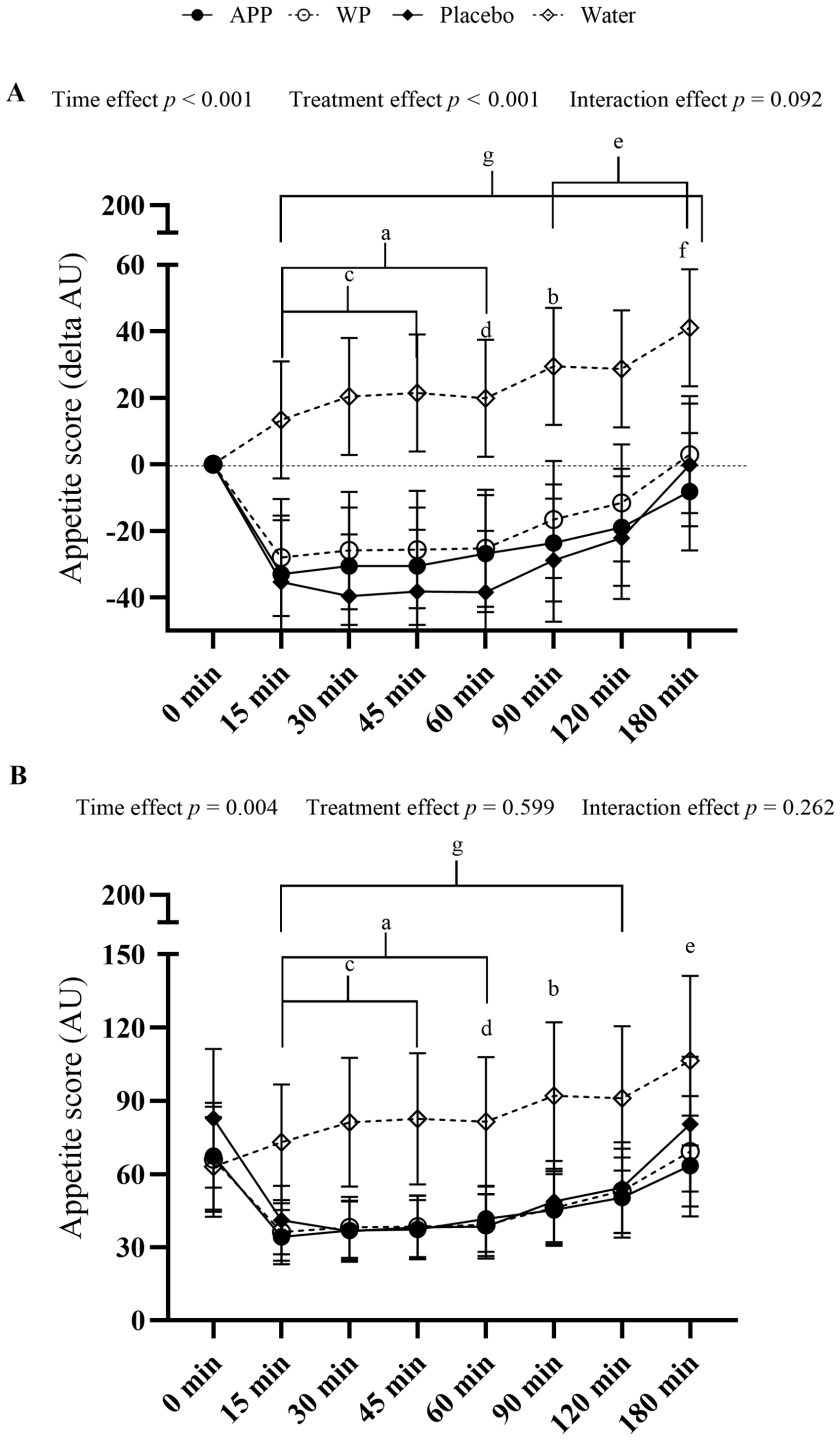
Appetite score following ingestion of almond protein (APP), whey protein (WP), placebo or water [*n* = 9 for all treatments, aside from placebo where *n* = 8] via composite of hunger, fullness, desire to eat, and urge to eat. Scores are presented as delta change from baseline values **(A)** and raw values **(B)**. *a* indicates that APP, WP, and placebo are all significantly different from their baseline value, *b* indicates that placebo is significantly different from baseline, *c* indicates APP, WP, and placebo are all significantly different from their 180 min value, *d* indicates WP, and placebo are significantly different from 180 min, *e* indicates water is significantly different from baseline, *f* indicates water is significantly different from 15 min, *g* indicates significant difference between water and all of APP, WP, and placebo. Statistical significance was set at a *p*-value <0.05 using linear mixed models.

**Figure 3:**
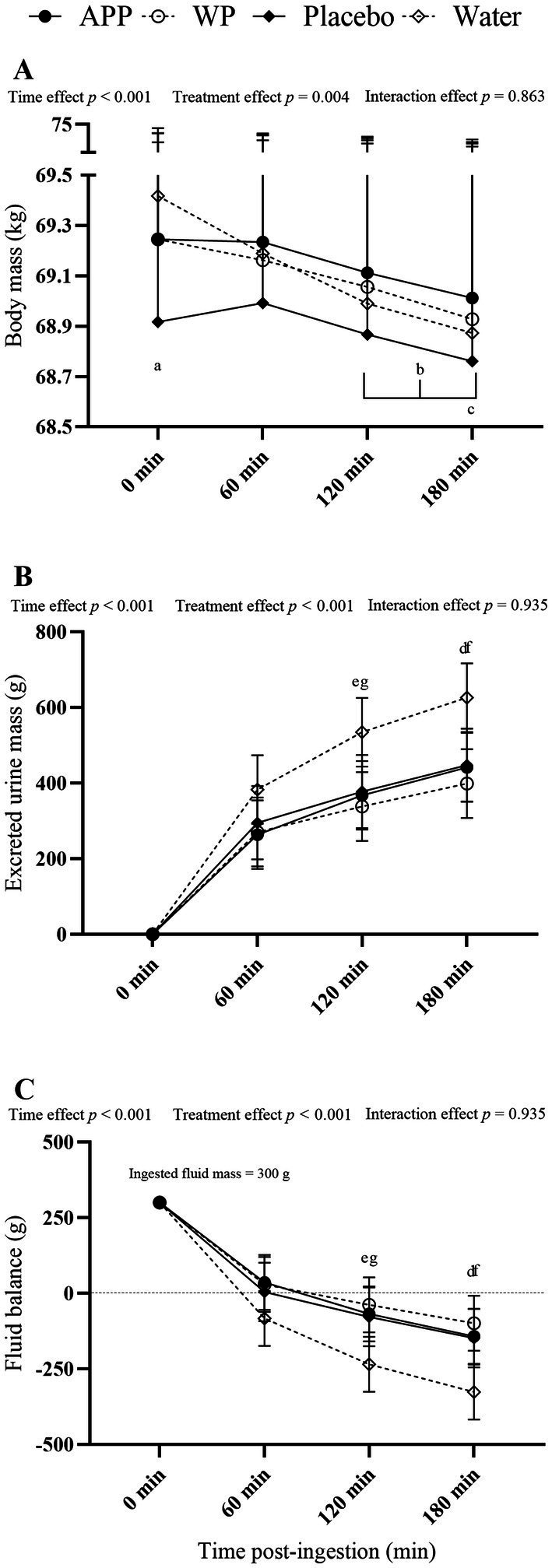
Body mass (**A**; kg), excreted urine mass (**B**; g) and fluid balance (**C**; g, calculated as fluid intake minus urine output) following ingestion of almond protein (APP), whey protein (WP), placebo or water [*n* = 9 for all treatments, aside from placebo where *n* = 8]. Data are presented as mean and confidence intervals over the 180 min post-ingestion period. Sample size was *n* = 9 for all treatments, except placebo (*n* = 8). *a* indicates significantly greater body mass for water compared to placebo, *b* indicates water significantly below baseline, *c* indicates WP significantly below baseline, *d* indicates APP, placebo and water significantly elevated above 60 min for urine mass (below 60 min for fluid balance), *e* indicates water significantly elevated above 60 min for urine mass (below 60 min for fluid balance), *f* indicates water significantly higher than the other three treatments for urine mass (below for fluid balance), *g* indicates water significantly above WP for urine mass (below WP for fluid balance). Statistical significance was set at a *p*-value <0.05 using linear mixed models. NB: the *y*-axis of Figure 2A was adjusted to improve visualisation of between-treatment differences while preserving error bar limits.

**Figure 4:**
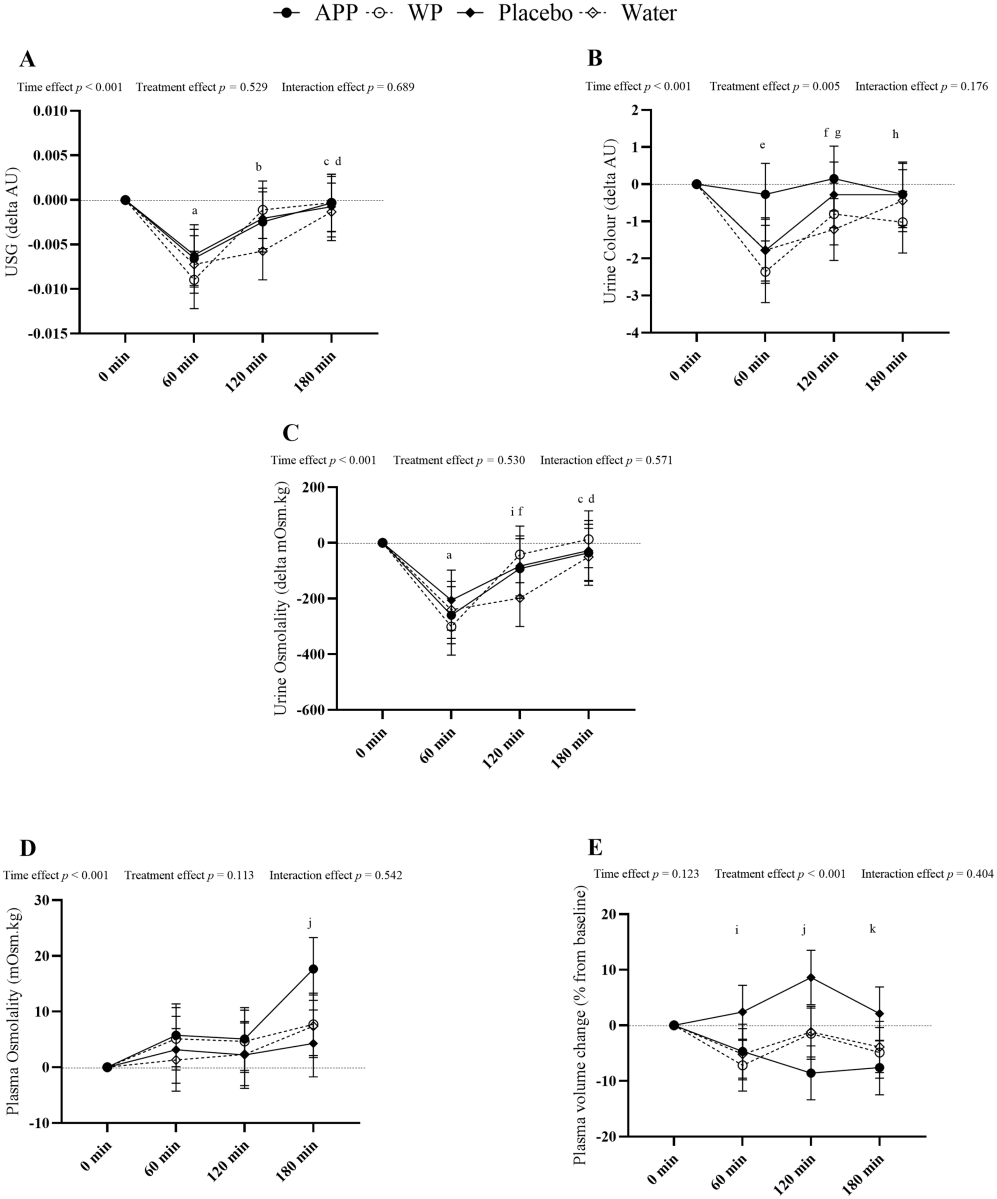
Delta values for urine specific gravity (USG, **A**; arbitrary units), urine colour (**B**; arbitrary units), urine osmolality (**C**; mOsm.kg), plasma osmolality (**D**; mOsm.kg) and plasma volume change (**E**; % change from baseline) following ingestion of almond protein (APP), whey protein (WP), placebo or water [*n* = 9 for all treatments, aside from placebo where *n* = 8]. Data are presented as mean and confidence intervals over the 180 min post-ingestion period. *a* indicates all treatments significantly lower than baseline, *b* indicates APP, WP, and placebo significantly higher than 60 min, *c* indicates all treatments significantly higher than 60 min, *d* indicates water significantly higher than 120 min, *e* indicates WP, placebo, and water significantly lower than baseline, *f* indicates water lower than baseline, *g* indicates WP and placebo significantly higher than 60 min, *h* indicates water significantly higher than 60 min, *i* indicates APP and WP significantly higher than 60 min, *j* indicates APP significantly higher than all prior times. Statistical significance was set at a *p*-value <0.05 using linear mixed models.

Timecourse outcomes were analysed using LMMs with treatment, time and their interaction included as fixed effects, session number included as a covariate, and participant as a random intercept. Treatment, time and treatment-by-time effects were assessed using joint Wald tests, and estimated marginal means with 95% confidence intervals were derived for visualisation and summary statistics. Where relevant, Sidak-adjusted pairwise comparisons were conducted to compare treatments within time points and time points within treatments to explore interaction and temporal patterns. To illustrate post-ingestion trajectory responses, LMMs were also run on delta values calculated relative to baseline. This approach was used to account for between-participant variability in absolute values and to visualise the treatment-related changes over time, and was applied only to variables where baseline was not inherently incorporated into post-ingestion values (e.g., USG, urine colour, urine osmolality, plasma osmolality, appetite score). Accordingly, delta models have been presented in Figures 2–4, with raw models presented in Supplementary File 2. Note for all urine analyses, waking urine sample outcomes were not included within LMMs, given their collection prior to arrival at the lab. These outcomes have been reported with other descriptives of pre-intervention hydration status (BIA outcomes) as mean and SDs (Table 2).

**Table 2:** Perceived responses to ingestion and ratings of protein drinks or water with regards to blinding efficacy, taste tolerability, appetite and gastrointestinal symptoms.

Outcome measure	Measurement time	Treatment						Treatment effect
		APP	WP	Placebo	Water	Non-water	All	*p*-value
Supplement ratings and identifications								
Supplement identification accuracy (%)	15 min	22%	22%	22%	100%	23%	43%	
	180 min	22%	22%	22%	100%	23%	43%	
Supplement identification confidence	15 min	14 (0–32)	8 (0–25)	14 (0–40)	50 (50–50)^a^	12 (0–40)	22 (0–50)	<0.001
	180 min	14 (0–35)	11 (0–30)	14 (0–40)	47 (25–50)^a^	13 (0–40)	22 (0–50)	<0.001
Supplement likability	15 min	4 (1–7)	4 (1–7)	5 (1–8)	5 (3–7)	4 (1–8)	5 (1–8)	0.232
Supplement taste		4 (1–7)	4 (1–7)	5 (1–8)	5 (3–7)	5 (1–8)	5 (1–8)	0.477
Supplement opportunity		6 (3–9)	6 (2–10)	5 (2–9)	3 (1–5)^	6 (2–10)	5 (1–10)	<0.001
Other perceived responses to supplementation								
Hunger iAUC	0–180 min post-ingestion	−362 (−1,341, 618)	−258 (−1,238, 721)	−765 (−1745, 215)	1897 (917, 2,876)^a^			0.001
Fullness iAUC		1,301 (192, 2,410)	1,397 (288, 2,506)	1,299 (190, 2,408)	−688 (−1796, 421)^			0.023
Desire to eat iAUC		−883 (−1,502, −263)	−588 (−1,208, 32)	−1,053 (−1,672, −433)	1,103 (483, 1722)^a^			<0.001
Urge to eat iAUC		−1,023 (−1,650, −397)	−369 (−996, 257)	−1,055 (−1,681, −429)	888 (261, 1,514)^a^			<0.001
Appetite score iAUC		−3,568 (−6,257, −880)	−2,613 (−5,301, 76)	−4,172 (−6,860, −1,483)	4,574 (1886, 7,262)^a^			<0.001
Severity of GIS post ingestion		4.0 (0.8, 7.9)	3.3 (0.5, 6.1)	4.0 (−0.4, 8.4)	0.6 (−0.6, 1.7)^a^			0.036

a Pairwise comparisons indicate this treatment was significantly greater than all others (*p* < 0.05). ^ Pairwise comparisons indicate this treatment was significantly lower than all others (*p* < 0.05). Values reported under “Supplement ratings and identifications” are presented as mean (range), except supplement identification accuracy which is reported as % only. Values reported under “Other perceived responses to supplementation” are presented as mean (95% confidence intervals).

All main effects for treatment, time and their interaction are presented visually in Figures 2–4. Statistical comparisons between treatments (e.g., APP vs. WP for Appetite iAUC), and between times within treatments (e.g., baseline vs. 180 min for APP for urine colour) are described in subsequent sections, with *p*-values reported in Supplementary File 4 and Tables 2, 3. Where comparisons between times within treatments do not include baseline (e.g., 60 min vs. 180 min for APP for body mass), *p*-values of relevance are reported in the results text. Pairwise comparisons for hunger, fullness, desire to eat and urge to eat have not been included to maximise readability of Supplementary File 4. All comparisons in these models returned p-values over 0.05, and primary focus of these outcomes is reflected in the appetite models to be discussed.

**Table 3:** Waking urine sample analytes, and baseline bioelectrical impedance analysis metrics prior to ingestion of experimental treatments.

Pre-intervention hydration status indicator		Treatment				Treatment effect
Source	Outcome	APP	WP	Placebo	Water	*p*-value
Waking urine sample	USG (ratio)	1.018 (0.008)^a^	1.014 (0.005)	1.013 (0.005)	1.014 (0.005)	0.002
	Colour (AU)	4.6 (0.9)	3.9 (1.5)	4.3 (0.8)	3.5 (1.1)	0.054
	Osmolality (mOsm.kg)	597.81 (261.21)^a^	442.78 (164.58)	430.43 (164.48)	450.75 (127.68)	0.004
BIA	Body mass (kg)	69.2 (14.4)	69.2 (14.4)	66.0 (12.3)^	71.2 (14.2)	<0.001
	Body fat (%)	37.3 (8.7)	36.6 (8.7)	35.9 (8.2)	37.3 (8.3)	0.352
	Total body water (kg)	31.0 (2.6)	31.4 (2.6)	30.4 (2.0)	32.0 (2.4)	0.063

a Pairwise comparisons indicate this treatment was significantly higher than all other treatments (*p* < 0.05). ^ Pairwise comparisons indicate this treatment was significantly lower than all other treatments (*p* < 0.05). APP, almond protein powder; WP, whey protein; USG, urine specific gravity; BIA, bioelectrical impedance analysis. All values reported here are presented as mean (standard deviation).

## Results

A final sample size of *n* = 9 was achieved, satisfying *a priori* power calculations (40). The nine participants who completed testing were of a mean (± SD) age 60 ± 4 years, completed menopause at 52 ± 4 years of age, rated their current physical activity participation between “a little active” and “fairly active” upon screening, and reported consuming nuts or nut products “3–4 times per week” on average. Washout period between experimental testing sessions was 7.9 ± 4.9 days, with a minimum of 3 days and a maximum of 21 days according to participant availability. Supplementation commenced at a mean time of 08:05, with the earliest and latest commencement times being 07:25 and 09:00, respectively, (no significant difference in supplementation commencement time between treatments, treatment effect *p* = 0.077). Of 36 total scheduled testing sessions, one session (placebo) was incomplete due to difficulty drawing blood.

### Blinding efficacy

Across 35 completed sessions, participants correctly identified the administered supplement in 15 (43%), were “not sure” in 11 (31%), and incorrect in 9 (26%). Excluding water, participants correctly identified APP, WP, or placebo in 6 of 26 sessions (23%), were incorrect in 9 (35%), and unsure in 11 (42%). Of the 15 correct identifications overall, 9 (60%) were for water, which was correctly identified in 100% of sessions. Confidence ratings reflected this pattern, with participants significantly more confident in identifying water than the other three treatments at both 15 (APP, WP and placebo *p* < 0.001 vs. water) and 180 min post-ingestion (APP, WP and placebo *p* < 0.001 vs. water, Table 2). Confidence in identifying APP, WP, and placebo was low, averaging 8–14 out of 50, whereas all participants correctly identified water with the maximum confidence score of 50.

### Appetite

For delta appetite scores, significant main effects of treatment and time were present (Figure 2; Supplementary File 4), while a significant treatment effect was not present for raw models. All non-water treatments were associated with reduced appetite from baseline to 15, 30, 45, and 60 min post-ingestion, which also extended to 90 min for placebo. Contrastingly, water was associated with elevated appetite compared to baseline at all 90–180 min times. For each of the non-water treatments, greatest suppression of appetite occurred within the first 30 min post-ingestion. Scores progressively returned toward baseline, with values approaching baseline by 180 min, indicating that the appetite response had largely subsided by the end of testing. In contrast, water showed a gradual increase in appetite score above baseline over time, with the highest values observed at 180 min. Each of the non-water treatment appetite scores were significantly lower than their respective 180 min value for both raw and delta models at 15 (raw models; APP *p* = 0.007, WP *p* = 0.005, placebo *p* = 0.006. Delta models; APP *p* = 0.022, WP *p* = 0.004, placebo *p* = 0.002), 30 (raw models; APP *p* = 0.015, WP *p* = 0.009, placebo *p* = 0.002. Delta models; APP *p* = 0.038, WP *p* = 0.008, placebo *p* = 0.001) and 45 min (raw models; APP *p* = 0.017, WP *p* = 0.009, placebo *p* = 0.003. Delta models; APP *p* = 0.038, WP *p* = 0.008, placebo *p* = 0.001), with this maintained at 60 min for WP (raw *p* = 0.011, delta *p* = 0.009) and placebo (raw *p* = 0.003, delta *p* = 0.001). In exploratory pairwise comparisons, all three non-water treatments were associated lower appetite than water at all time points from 15 min to 120 min post-ingestion for raw models, and for all these times as well as 180 min post-ingestion for delta models The four appetite sub-domains (hunger, fullness, desire to eat, urge to eat) are presented visually in Supplementary File 3. Model comparisons revealed no consistent treatment, time or interaction effects across these sub-domains, though hunger demonstrated a significant treatment by time interaction; with no significant between-treatment differences at specific time points. These domains of appetite were also assessed for iAUC (Table 3), where water was associated with a significantly higher hunger, desire to eat, urge to eat, and appetite score, with no specific differences between treatments for fullness despite a significant main effect of treatment at the model level.

### Participant ratings of likability and gastrointestinal symptoms

Likability scores ranged between 4 and 5 across all treatments, both individually and when combined, corresponding to participants reporting they “somewhat” to “fairly” liked the taste of administered treatments in this study (Table 2). For the three non-water treatments, mean ratings for willingness to seek out the drink ranged from 5 to 6, aligning with responses of “I would drink this if available but would not go out of my way” to “I do not like it but would drink it on an occasion.” Water was significantly preferred on this scale compared with APP (*p* = 0.001) and WP (*p* < 0.001), with a mean rating of 3, corresponding to “I would frequently drink this.”

Due to a high frequency of zero ratings across post-ingestion time points, a time-course analysis of GIS was not feasible; therefore, total session symptom severity values are presented as the sum of all symptoms in a given session. Overall GIS severity was significantly greater for PLA compared with water (Table 2; Supplementary File 4), with no other differences between treatments detected. Across 35 total sessions, 18 (51%) included no post-ingestion GIS. Of 245 distinct post-ingestion GIS assessments, 187 (76%) recorded a score of 0 for all symptoms. When GIS were reported, the most frequently reported symptoms were belching (26 occurrences; 11%), heartburn and bloating (13 occurrences each; 5%). The total severity scores for these GIS across all sessions were 36 for belching (maximum possible severity = 2,450), 20 for heartburn, and 16 for bloating. Several symptoms (stomach pain, regurgitation, vomiting, loose stool, diarrhoea, blood in stool) were not reported at any time point, and no participant reported a severe symptom (GIS rated at a severity ≥5/10) following any treatment.

### Pre session hydration indicators

Waking USG and urine osmolality varied across conditions (Table 3), with significantly higher values observed prior to APP sessions as compared to WP (USG *p* = 0.011, urine colour *p* = 0.015), placebo (USG *p* = 0.005, urine colour *p* = 0.009) and water (USG *p* = 0.009, urine colour *p* = 0.027). At baseline, body mass assessed using regular scales differed significantly between water and placebo sessions (*p* = 0.049, Figure 3), while BIA-assessed body mass was significantly lower in the placebo sessions as compared to the other three treatments (APP *p* = 0.017, WP *p* = 0.001, placebo *p* < 0.001). To account for these pre-session differences, hydration outcomes to follow are presented using both raw values and change from baseline (delta) models.

### Hydration indices

#### Fluid balance, body mass and urine mass

Within the water treatment, body mass was significantly below baseline at both 120 min and 180 min (Figure 3). Body mass was significantly below baseline at the 180 min mark for WP. For excreted urine mass, each of APP, placebo and water treatments were significantly above the 60 min mark at 180 min, while the water condition achieved this by 120 min. Exploratory between-treatment differences were identified between water and all three treatments at the 180 min mark. For fluid balance, each of APP (*p* = 0.007), placebo (*p* = 0.028) and water (*p* < 0.001) treatments were significantly below the 60 min mark at 180 min, while the water condition achieved this by 120 min (*p* = 0.021).

#### USG

In delta models for USG, only a main effect for time was detected (Figure 4; Supplementary File 4). Delta analyses showed decreases from baseline to 60 min, and subsequent increases from 60 to 180 min for all treatments (APP *p* = 0.002, WP *p* < 0.001, placebo *p* = 0.010, water *p* = 0.003). USG for APP, WP and placebo was higher at 120 min than 60 min (APP *p* = 0.045, WP *p* < 0.001, placebo *p* = 0.049), with water also higher at 180 than 120 (*p* = 0.026). Raw models showed significant main effects for treatment and time, with no interaction (Supplementary File 2). USG decreased from baseline to 60 min for all treatments and was higher at 120 than at 60 min for WP and placebo. USG remained lower than baseline at 120 min for water only. At 180 min, USG for all treatments was higher than for 60 min (APP *p* = 0.002, WP *p* < 0.001, placebo *p* = 0.010, water *p* = 0.003). Water was also elevated at 180 compared to 120 (*p* = 0.026).

#### Urine colour

In delta analyses for urine colour, main effects for treatment and time were detected, with no interaction (Figure 4; Supplementary File 4). WP, placebo and water were lower at 60 min than baseline, with water remaining lower at 120 min. WP and placebo were higher at 120 than at 60 min (WP *p* = 0.004, placebo *p* = 0.008), with water significantly higher at 180 min than at 60 (*p* = 0.013). APP did not significantly differ from baseline at any post-ingestion time. Raw models returned significant main effects for treatment and time, and no interaction (Supplementary File 2). WP, placebo and water significantly decreased from baseline to 60 min, and water remained lower than baseline at 120 min. WP and placebo increased from 60 to 120 min (WP *p* = 0.004, placebo *p* = 0.009) and no longer differed from baseline. Water was higher at 180 min than at 60 (*p* = 0.014).

#### Urine osmolality

Delta analyses for urine osmolality showed no treatment or interaction effects, with significant time effects present (Figure 4; Supplementary File 4). All treatments decreased from baseline to 60 min, and increased at 180 min compared to 60 min (APP *p* < 0.001, WP *p* < 0.001, placebo *p* = 0.009, water *p* = 0.003). APP and WP were higher at 120 than 60 min (APP *p* = 0.010, WP *p* < 0.001), and water remained lower at 120 min as compared to baseline, before increasing from 120 to 180 min (*p* = 0.019). Raw models showed significant main effects for treatment and time, with no interaction (Supplementary File 2). All treatments decreased from baseline to 60 min. Water remained lower at 120 min, whereas APP and WP were greater at 120 than 60 min (APP *p* = 0.033, WP *p* < 0.001). All treatments were higher at 180 than 60 min (APP *p* = 0.001, WP *p* < 0.001, placebo *p* = 0.009, water *p* = 0.003), and water was higher at 180 than 120 min (*p* = 0.022).

#### Plasma osmolality

Delta analyses for plasma osmolality returned significant effect for time, but with no treatment or interaction effects (Figure 4; Supplementary File 4). Only APP at 180 min was different from all prior time points (60 min *p* < 0.001, 120 min *p* = 0.003, 180 min *p* = 0.002). Raw models retained significant main effects time with no interaction (Supplementary File 2), but treatment effects were significant. Only APP was elevated at 180 min as compared with all prior time points for that treatment (60 min *p* = 0.003, 120 min *p* = 0.045, 180 min *p* = 0.034), with no other differences detected.

#### Plasma volume

Plasma volume change (PVC) showed a significant main effect for treatment, with no main effect for time and no interaction (Figure 4; Supplementary File 4). Exploratory contrasts indicated that PVC was greater for placebo than for WP at 60 min (*p* = 0.030), and greater than all three other treatments at 120 min (APP *p* < 0.001, WP *p* = 0.019, water *p* = 0.027), and greater than APP at 180 min (*p* = 0.037). No within-treatment time differences were detected. Baseline comparisons are constrained by the calculation method; whereby baseline PVC values are fixed at zero.

#### Beverage hydration index

**Figure 5:**
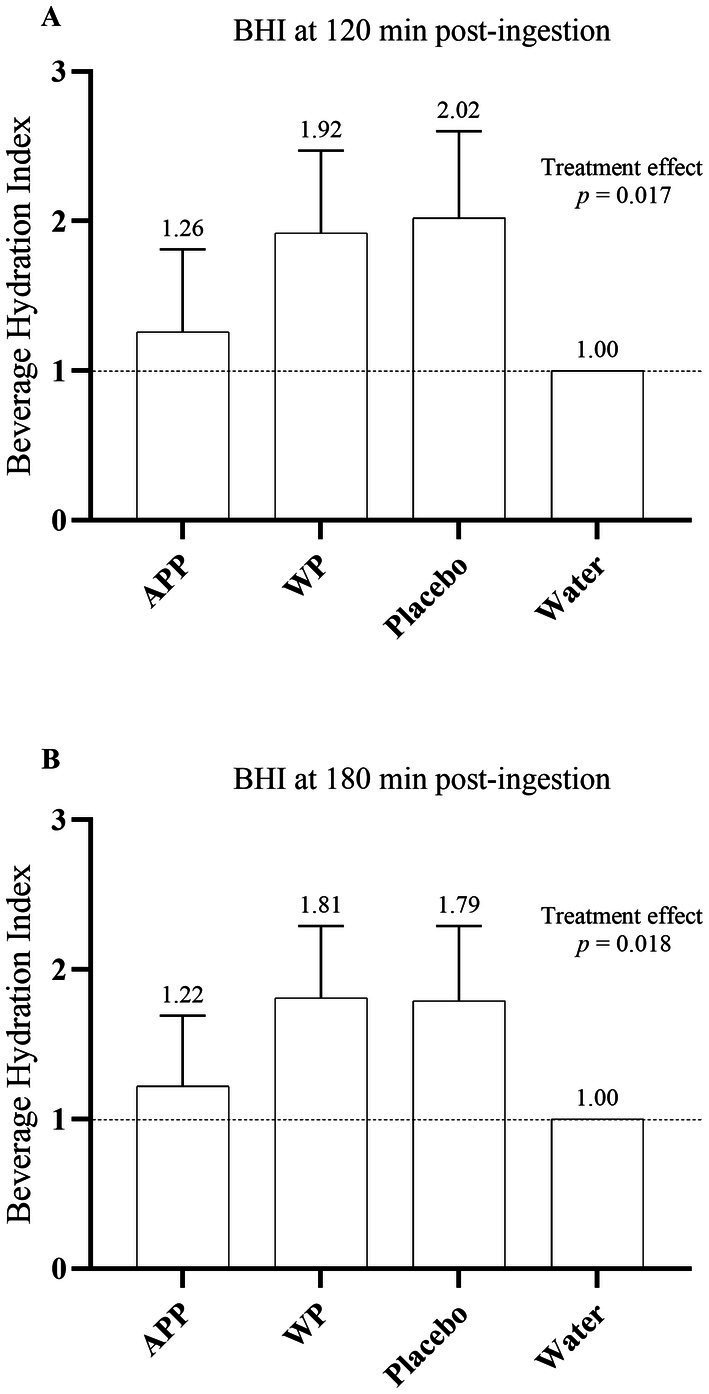
Beverage hydration index 120 min **(A)** and 180 min **(B)** after ingestion of almond protein (APP), whey protein (WP), placebo or water [*n* = 9 for all treatments, aside from placebo where *n* = 8]. Values are calculated as the ratio of cumulative urine mass following ingestion of water to that following ingestion of each experimental drink over the same time. Data are presented as mean and confidence intervals, with statistical significance set at a *p*-value <0.05 using linear mixed models. NB: T values for water are standardised to precisely 1.0 for all participant sessions, so no variation is presented.

BHI differed between treatments at both 120 and 180 min post-ingestion (*p* = 0.0165 and 0.0180, respectively; Figure 5), though post-hoc between-treatment comparisons did not return any significant differences between any combination of treatments at either time (all *p* > 0.05). Within-treatment comparisons between 120 and 180 min revealed no significant changes in BHI for APP (*p* = 0.8631), WP (*p* = 0.8432) or placebo (*p* = 0.6544).

## Discussion

Key findings of this study were that both APP and WP lowered appetite with minimal GIS and greater short-term fluid retention compared to water. Contrastingly, the two protein-containing beverages followed approximately similar magnitude and temporal patterns for outcomes relating to appetite, GIS and hydration. Of note, participants did not distinguish between nutrient-containing beverages with regards to taste preferences, confirming blinding efficacy and supporting the rigour of subjective measurements in this study. These findings point to the utility of APP by postmenopausal women to improve protein and fibre intake and to facilitate acute hydration.

### Appetite

Present findings demonstrated a clear effect of nutrient-containing beverage ingestion on subjectively rated appetite, which subsided over three hours. The presence of a treatment effect in delta but not raw models suggests that differences were driven by change from baseline rather than absolute appetite values, primarily due to contrasts between water and the three other treatments. Given subjective ratings of appetite can be susceptible to inherent variability (50, 51), this treatment effect is particularly telling. All nutrient-containing beverages reduced appetite below baseline within the first 60 min, with responses returning toward baseline by 180 min, whereas water showed a gradual increase in appetite over time. Post-ingestion iAUC analyses reinforced this pattern, with nutrient-containing beverages reducing total appetite and its components compared with water. These findings indicate greater appetite suppression with nutrient-containing beverages, likely reflecting combined effects of energy provision and specific nutrient sensing within the gastrointestinal tract (52, 53).

The early reduction in appetite observed with APP, WP and placebo (compared to water and to baseline) is consistent with established mechanisms underpinning protein or carbohydrate-induced appetite suppression (18, 19). Acute gastric distension can contribute to transient appetite suppression via gastric mechanoreceptor signalling (54); however, as beverage volume was matched across all treatments, this is unlikely to explain the greater suppression seen with nutrient-containing beverages compared with water. Instead, nutrients delivered to the small intestine may stimulate gut-derived satiety signalling (e.g., GLP-1), which can slow gastric emptying and prolong distension, thereby augmenting appetite suppression (55). The transient responses observed may also reflect the liquid format, which tends to produce shorter-lived effects than solid foods due to reduced oral processing and faster gastric emptying (56, 57), although this remains speculative in the absence of a solid food comparator. The moderate protein dose may also have been sufficient to elicit acute endocrine responses, but insufficient to sustain appetite suppression beyond the early postprandial period (17, 58).

No differences were observed between APP and WP despite compositional differences (Table 1). WP is rapidly digestible and rich in branched-chain amino acids (59), whereas APP provides a broader matrix including fat and fibre that may also influence hormonal appetite signals (60, 61). However, when protein dose, energy, and volume are matched in liquid form, protein source may have limited influence on acute appetite relative to overall nutrient exposure (18, 62). This aligns with studies showing robust appetite responses to WP versus water or carbohydrate controls (63, 64), and comparable effects between dairy and plant proteins under matched conditions (65, 66). This is particularly noteworthy because APP, despite being a plant-based protein source that would typically be expected to elicit a weaker satiety response than WP, achieved comparable appetite outcomes, possibly due to a higher fibre and fat content, highlighting the functional capability of both protein sources as ingredients suitable for appetite regulation. The comparable APP and WP responses may, in part, reflect postmenopausal physiological changes, as oestrogen decline may influence gastrointestinal function and regulation of appetite-related hormones, including cholecystokinin and peptide YY, which respond to dietary protein and fat (67). However, this interpretation remains speculative, as the present study did not include premenopausal women or age-matched men for comparison. The placebo also induced similar appetite suppression, likely due to rapidly absorbed maltodextrin delivering a substantial carbohydrate load to the small intestine, stimulating gut-derived appetite regulation despite minimal protein content (68).

Collectively, APP, WP, and placebo elicited comparable, short-lived appetite suppression versus water, largely within the first 60 min post-ingestion. Differences from baseline in delta analyses highlight the importance of accounting for baseline variability in subjective appetite ratings. APP therefore appears broadly comparable to WP for acute appetite regulation in liquid form. Future work should incorporate objective biomarkers, alternative doses or formats, and longer-term intake assessments (69).

### Other perceived responses

The GIS burden in this study was minimal, indicating that all treatments were well tolerated in this cohort. The absence of any severe complaints suggest that ingestion of these protein-containing beverages did not meaningfully disrupt gastrointestinal comfort. This is consistent with prior works on WP, which generally induces low GIS incidence in non-clinical groups when consumed in liquid form (70, 71). The present findings extend this tolerability profile to APP under similar conditions. The highly skewed distribution of GIS data, characterised by a pronounced number of zero values, restricted the feasibility of timecourse analyses and limited statistical sensitivity to detect small between-treatment differences. However, this skew is informative in that it indicates that any physiological perturbation was negligible in magnitude. Mechanistically, the comparable GIS profiles of APP and WP are not unexpected. Both were administered at common protein doses within a liquid matrix matched for fluid volume and energy content, factors which can influence gastric emptying rate and subsequent GIS. In the absence of large differences in lactose, poorly absorbed carbohydrates, or fermentable substrates (72), there is limited rationale to have anticipated divergent GIS outcomes for these treatments in this study. Even in considering the relatively high fibre content of APP, whereby the 13 g ingested by the participants is approximately half the recommended daily intake for adult women (73), participants did not report severe or frequent GIS.

Likability ratings were also comparable between APP and WP, suggesting that neither protein source imposed a sensory disadvantage when delivered in a standardised format. In liquid formulations, differences in protein source can be attenuated through flavour masking and matched viscosity, limiting the potential perceptible differences in taste and mouthfeel (74). The broadly similar “willingness-to-consume” responses supports the practical feasibility of APP as an alternative to WP from a consumer acceptability perspective.

Blinding efficacy strengthens interpretation of subjective outcomes in this study. Although water was readily identifiable, correct identification of the other three treatments occurred in only 23% of sessions, below even a random guess accuracy rate amongst three options (33%). This, combined with low identification confidence for the three non-water treatments, indicates effective blinding between those beverages in this study. Such blinding is particularly important here given the subjective nature of GIS and appetite measures, which can be susceptible to expectancy effects (75).

### Hydration

Across hydration outcomes, all beverages showed an expected early dilution at 60 min, followed by progressive reconcentration towards 120–180 min, with no treatment by time interactions. Significant treatment effects were observed for most hydration markers, though these were largely driven by water differing from the other beverages rather than differences amongst APP, WP and placebo. These patterns indicate a shared, physiologically driven diuresis and recovery response largely independent of beverage composition (76). Similar kinetic trajectories and parallel curves suggest beverage composition influenced the magnitude of fluid retention without altering overall hydration patterns.

Comparison with established references ranges suggested participants were generally euhydrated at waking and baseline, although hydration thresholds specific to postmenopausal women remain limited. Mean waking values for USG, urine colour, urine osmolality and plasma osmolality were below commonly proposed dehydration thresholds derived from healthy adults (77), athletes (78) and older adults (79). However, these comparisons should be interpreted cautiously, given differing populations and sampling protocols in those prior works. Baseline values for USG, urine colour and urine osmolality were not exceeded at any post-ingestion time point, indicating maintenance of improvement of hydration status following beverage ingestion.

BHI values exceeding 1.0 indicate greater fluid retention than water (39), with previous works showing full-fat milk can achieve values of ~1.5 at 120 min post-ingestion (39). In the present study, treatment effects for BHI at 120 and 180 min suggest beverage composition influenced urine excretion relative to water, although pairwise differences were absent. This may reflect both statistical factors, because BHI is expressed relative to fixed water values of 1.0, and physiological factors, as the protein-containing beverages were compositionally similar.

Mechanistically, protein-containing beverages may enhance fluid retention relative to water through slowed gastric emptying (80, 81), insulin-mediated sodium retention (82), and increased plasma oncotic pressure (39). Potential APP-WP differences could arise from digestion kinetics, amino acid profile (83, 84), and APP fibre and fat content (85). Despite these theoretical expectations, APP, WP and placebo produced broadly similar fluid balance and BHI responses. This may reflect the modest fat content of APP and the dominant influence of sodium concentration and osmolality on short-term fluid retention (86, 87). Additionally, APP’s dispersed liquid matrix may reduce matrix-related effects compared with intact almonds (85, 88). The modest decline in BHI from 120 to 180 min across non-water beverages suggests any hydration advantage relative to water was transient and diminished as renal equilibration occurred. These findings indicate physicochemical characteristics and total fluid volume exerted a greater influence on short-term hydration than protein source alone. Attenuation of treatment effects in delta models further supports this interpretation, suggesting largely parallel responses once baseline variation was accounted for.

Urinary and systemic hydration markers also diverged. Urine osmolality showed clear time-dependent changes and contributed to treatment effects, whereas plasma osmolality remained relatively stable, indicating renal responses without meaningful disruption to systemic osmolality (89). Given the tight physiological regulation of plasma osmolality (90), modest beverage composition differences under resting conditions may be insufficient to alter systemic homeostasis compared with more extreme perturbations [e.g., extreme heat, humidity or ingestion of very large fluid boluses (91, 92)]. Thus, acute beverage effects appeared primarily through urinary handling rather than systemic shifts.

These findings should be interpreted within the context of the postmenopausal cohort. Menopause is associated with altered body water distribution (33), renal concentrating capacity (93), and hormonal regulation of fluid balance (32, 35), which may attenuate beverage-specific effects relative to younger populations. The similar trajectories observed between APP, WP and water likely reflect tight homeostatic regulation of plasma osmolality and plasma volume. Minimal differences in plasma osmolality and the absence of consistent plasma volume shifts reinforce the interpretation that hydration responses were predominantly time-dependent rather than beverage-dependent. From a practical perspective, APP, WP and water appear to provide similar short-term hydration under resting conditions in postmenopausal women.

### Practical relevance for postmenopausal women

The findings reported in this study have practical implications for postmenopausal women, who face elevated risk of sarcopenia and age-related reductions in thirst perception and renal function associated with dehydration. Adequate protein intake is vital for maintaining muscle mass and function, and protein-containing beverages provide a convenient option to support other dietary protein intake. While this study did not assess markers of protein metabolism or muscle function, these data indicate that APP and WP can be consumed in liquid form without compromising short-term hydration, gastrointestinal comfort or palatability. Both protein sources demonstrated comparable appetite and tolerability profiles as well as hydration responses, suggesting that either could be used to support protein intake while contributing to overall fluid intake. These outcomes highlight the feasibility of incorporating liquid protein supplements into the dietary habits of postmenopausal women for dual purposes; nutrition and hydration.

### Limitations and future directions

Some limitations should be considered when interpreting these findings. The placebo treatment was not physiologically inert with respect to hydration indices, as all non-water treatments contained almond milk, chocolate flavouring and an additional component (almond protein, whey protein or maltodextrin for placebo). This placebo treatment primarily serves as a control arm for an associated and yet unpublished study, with water acting as the main comparator in the present study. Post-ingestion urine sampling timing (once per hour after baseline) may have reduced statistical sensitivity in detecting transient or time-dependent differences in hydration and appetite. Future studies incorporating more frequent sampling, extended monitoring periods, and habitual protein supplementation may provide additional insight into the temporal dynamics and real-world applications of these findings. Some baseline variation in body composition was observed, though pre-arrival water standardisation, consistent baseline urinary markers and the use of delta modelling sought to mitigate this issue. These considerations highlight that the present findings reflect acute, short-term responses and may not fully extrapolate to habitual supplementation scenarios or other populations. While power calculations were completed and reported *a priori*, and the calculated sufficient sample size was met for the primary physiological outcomes, the sample size of nine participants limits the strength of inference that can be made for subjective appetite and GIS outcomes. Accordingly, these findings should be interpreted conservatively.

## Conclusion

Almond protein and whey protein both elicited transient decreases in appetite and greater short-term hydration compared with water in postmenopausal women, while demonstrating comparable responses to one another. Both protein sources produced brief subjective appetite reductions during the early post-ingestion period, with no notable differences thereafter. Hydration responses were largely characterised by parallel temporal patterns across assessed beverages, suggesting that composition influenced the magnitude of early responses relative to water without meaningfully altering overall trajectory of hydration. Collectively, these findings indicate that APP and WP provide comparable appetite and hydration responses while remaining well tolerated in postmenopausal women. From a practical perspective, these results support either protein source as a strategy to contribute to dietary protein intake on the basis of appetite and hydration considerations in this population.

## Data Availability

The datasets presented in this study can be found in online repositories. The names of the repository/repositories and accession number(s) can be found in the article/Supplementary material.

## Supplementary material

SUPPLEMENTARY FILE 1 Supplement ingredient details. Macronutrient, fibre, sodium and energy content of the ingredients that compose the four treatments within this investigation.

SUPPLEMENTARY FILE 2 Raw values for urine specific gravity (USG, **A**; arbitrary units), urine colour (**B**; arbitrary units), urine osmolality (**C**; mOsm.kg), and plasma osmolality (**D**; mOsm.kg) following ingestion of almond protein (APP), whey protein (WP), placebo or water [*n* = 9 for all treatments, aside from placebo where *n* = 8]. Data are presented as mean and confidence intervals over the 180 min post-ingestion period. *a* indicates all treatments significantly lower than baseline, *b* indicates water significantly lower than baseline, *c* indicates all treatments significantly higher than 60 min, *d* indicates WP and placebo significantly higher than 60 min, *e* indicates water significantly higher than 120 min, *f* indicates WP and placebo significantly lower than baseline, *g* indicates APP and WP significantly higher than 60 min, *h* indicates APP significantly higher than all prior times, *i* indicates placebo significantly higher than WP, *j* indicates placebo significantly greater than all three other treatments, *k* indicates placebo significantly greater than APP. Statistical significance was set at a *p*-value <0.05 using linear mixed models.

SUPPLEMENTARY FILE 3 Perceived changes in hunger **(A)**, fullness **(B)**, desire to eat **(C)** and urge to eat **(D)** following ingestion of almond protein (APP), whey protein (WP), placebo or water [*n* = 9 for all treatments, aside from placebo where *n* = 8]. Statistical significance was set at a *p*-value <0.05 using linear mixed models.

SUPPLEMENTARY FILE 4 *P*-values derived from models for appetite, other subjective variables, blood, urine, and body mass responses, including main effects, within-treatment changes from baseline and between-treatment differences at each time point following ingestion of almond protein, whey protein, placebo, or water.
