# Supplementary material for: Almond protein and whey protein beverages elicit comparable appetite regulation, gastrointestinal symptoms and hydration responses in postmenopausal women: a randomised crossover trial
Source: Front Nutr. 2026 Jul 28;13:1918989. doi: 10.3389/fnut.2026.1918989 (PMC13457626; doi:10.3389/fnut.2026.1918989)
Supplement: Supplementary file 1 [file Supplementary_file_1.DOCX]

| **Dietary Component** | **Composition of ingredients, at the quantity used in the total treatments** | | | | | |
| --- | --- | --- | --- | --- | --- | --- |
|  | Almond protein powder | Whey protein powder | Almond Milk | Water | Maltodextrin | Chocolate Flavouring |
| Manufacturer | Harris Woolf | Bulk Nutrients | Coles | Coles | Bulk Nutrients | Coles |
| Quantity (g) | 56 | 29.8 | 300 | 300 | 60 | 30 |
| Carbohydrate (g) | 16.7 | 1.0 | 2.5 | 0 | 57 | 24 |
| Protein (g) | 26.2 | 26.2 | 1.7 | 0 | 0 | 0 |
| Fat (g) | 7.3 | 0.3 | 4.5 | 0 | 0 | 0.7 |
| Fibre (g) | 10.0 | 0 | 0.6 | 0 | 0 | 2.4 |
| Sodium (mg) | 6.0 | 65 | 135 | 0 | 1 | 61 |
| Energy (kJ) | 1000 | 481 | 242 | 0 | 960 | 485 |
