# Supplementary material for: Almond protein and whey protein beverages elicit comparable appetite regulation, gastrointestinal symptoms and hydration responses in postmenopausal women: a randomised crossover trial
Source: Front Nutr. 2026 Jul 28;13:1918989. doi: 10.3389/fnut.2026.1918989 (PMC13457626; doi:10.3389/fnut.2026.1918989)
Supplement: Supplementary file 2 [file Supplementary_file_4.DOCX]

**Additional File 4 – Table 1.**

Main effects for treatment, time and interaction (treatment x time) for appetite, other subjective measurements, blood, urine and body mass measurements. Variables are defined within the Methods section of the manuscript. Significant effects are shown in bold (*p* < 0.05). Between-treatment comparisons (e.g. APP vs WP) are reported only for whole-session variables (e.g. appetite iAUC), while treatment comparisons for time-course models are presented in the subsequent Table. iAUC = incremental area under the curve, GIS = gastrointestinal symptoms, USG = urine specific gravity, APP = almond protein powder, WP = whey protein. Where effects were not assessed (e.g. time effect for whole-session variables), a dash (-) is shown.

**Additional File 4 – Table 2a.**

**Additional File 4 – Table 2b.**

P-values for timecourse comparisons are presented for within-treatment changes from baseline (e.g. APP vs baseline for each time point) and between-treatment differences at each time point (e.g. APP vs WP at 30 min). Variables are defined in the Methods section of the manuscript. Only significant effects are reported (*p* < 0.05); absent values indicate non-significant comparisons (these are available upon reasonable request from the corresponding author). A dash (-) indicates that no comparison was performed. Significant comparisons between time points that do not include baseline (e.g. 15 min vs 180 min for APP) are described in the main text Results section. For clarity, the table is separated into panels (a) and (b). Subdomains of appetite are not presented, due to absence of significant effects. USG = urine specific gravity, APP = almond protein powder, WP = whey protein, delta refers to relative change from baseline. Effects reported here are also visually presented in Figures 2-4.
